# Tankyrase inhibitors attenuate WNT/β-catenin signaling and inhibit growth of hepatocellular carcinoma cells

**DOI:** 10.18632/oncotarget.4455

**Published:** 2015-06-27

**Authors:** Li Ma, Xiaolin Wang, Tao Jia, Wei Wei, Mei-Sze Chua, Samuel So

**Affiliations:** ^1^ Asian Liver Center and Department of Surgery, Stanford University School of Medicine, Stanford University, Stanford, CA 94305, USA; ^2^ School of Pharmaceutical Sciences, Wuhan University, Wuhan, Hubei, 430072, China; ^3^ Guangdong Institute of Gastroenterology, Guangdong Provincial Key Laboratory of Colorectal and Pelvic Floor Diseases, The Sixth Affiliated Hospital, Sun Yat-Sen University, Guangzhou, Guangdong, 510655, China

**Keywords:** WNT/β-catenin signaling, hepatocellular carcinoma, tankyrase inhibitors, β-catenin/TCF complexes, targeted therapy

## Abstract

Deregulated WNT/β-catenin signaling contributes to the development of a subgroup of hepatocellular carcinoma (HCC), the second leading cause of cancer deaths worldwide. Within this pathway, the tankyrase enzymes (TNKS1 and TNKS2) degrade AXIN and thereby enhance β-catenin activity. We evaluate TNKS enzymes as potential therapeutic targets in HCC, and the anti-tumor efficacy of tankyrase inhibitors (XAV939, and its novel nitro-substituted derivative WXL-8) in HCC cells. Using semi-quantitative RT-PCR, we found significantly elevated levels of TNKS1/2 mRNA in tumor liver tissues compared to adjacent non-tumor livers, at protein levels only TNKS1 is increased. In HepG2, Huh7cells, siRNA-mediated knockdown suppression of endogenous TNKS1 and TNKS2 reduced cell proliferation, together with decreased nuclear β-catenin levels. XAV939 and WXL-8 inhibited cell proliferation and colony formation in HepG2, Huh7, and Hep40 cells (*p* < 0.05), with stabilization of AXIN1 and AXIN2, and decreased β-catenin protein levels. XAV939 and WXL-8 also attenuated rhWNT3A-induced TOPflash luciferase reporter activity in HCC cells, indicating reduced β-catenin transcriptional activity, consistent with decreased nuclear β-catenin levels. *In vivo*, intra-tumor injections of XAV939 or WXL-8 significantly inhibited the growth of subcutaneous HepG2 xenografts (*P* < 0.05). We suggest that tankyrase inhibition is a potential therapeutic approach for treating a subgroup HCC with aberrant WNT/β-catenin signaling pathway.

## INTRODUCTION

Hepatocellular carcinoma (HCC) is the sixth most common cancer, and second leading cause of cancer deaths worldwide [1], accounting for more than 745,000 deaths annually. Although predominant in sub-Saharan Africa and Asia, its incidence is increasing in the United States, where there are an estimated 33,000 new cases in 2014 [2]. Chronic infections with hepatitis B or hepatitis C virus (HBV or HCV), and chronic alcoholic consumption are well-established etiology factors of HCC [3]. About 40% of those diagnosed at an early stage are eligible for curative surgical resection and liver transplantation. Treatment of HCC is challenged by its heterogeneous molecular pathogenesis and complex disease background [4]. Sorafenib is the only FDA approved, first line therapy for patients with advanced HCC [5]; however, there is no second line therapy available for patients who do not respond to, or have developed resistance to sorafenib [6]. Although an increasing number of drug candidates are under preclinical/clinical investigations, many phase III clinical trials reported no significant survival benefit compared to sorafenib [7]. Thus, there is an unmet need for more potent targeted or adjuvant therapies to improve current HCC management.

The WNT/β-catenin pathway is essential during the developmental stage, and in maintaining normal cellular homeostasis, cell proliferation, differentiation, and pluripotency [8]. It is estimated that this pathway is deregulated in 50% of HCC cases [9]. In the inactive state, the effector molecule β-catenin is degraded and rendered inactive in the cytoplasm by the multi-protein destruction complex consisting of Adenomatous Polyposis Coli (APC), AXIN, and two kinases, Glycogen synthase kinase-3 (GSK3) and casein kinase (CK1)α/β. In the active state, binding of WNT protein ligands to the frizzled receptors triggers the downstream signaling cascade, causing the destruction complex to disassociate, and free β-catenin to accumulate in the cytoplasm and to translocate into the cell nucleus, where it interacts with TCF4/LEF to transcriptionally activate downstream target genes [10], such as c-myc [11] and Cyclin D1 [12], thereby promoting uncontrolled cell proliferation. Numerous clinical investigations have reported elevated levels of canonical WNT ligands [13, 14], frizzled receptor 7 [15], and co-receptor LRP6 [16] in HCC tissues compared to adjacent or normal liver tissues, suggesting hyperactivated Wnt/β-catenin pathway in HCC cells.

The WNT/β-catenin pathway is considered a particularly difficult target for molecular interventions, because of its many non-obvious enzyme targets which may disrupt necessary and beneficial biological processes [17]. Despite this, a high-throughput chemical screen identified small molecule inhibitors of this pathway, specifically XAV939, which was characterized as a potent inhibitor of tankyrase 1 (TNKS1/PARP5A) and tankyrase 2 (TNKS2/PARP5B) [18]. Both TNKS1 and TNKS2 destabilize the levels of AXIN1 and AXIN2, thereby reducing β-catenin degradation with consequent hyperactivation of WNT/β-catenin signaling [18]. Tankyrase inhibitors are thereby expected to stabilize AXIN levels leading to reduced β-catenin levels and activity. Tankyrases are members of the poly(ADP-ribose) polymerase (PARP) family, and regulate post-translational modification and degradation of target proteins *via* parsylation. Thus, these enzymes also promote mitosis and telomerase function. As such, tankyrases are promising candidate targets for anti-cancer molecular therapies [19], and they hold particular promise for HCC since their over-expression has been previously detected under the pathogenic conditions of cancer, tissue fibrosis, and viral infection [20]. Indeed, recent studies of the TNKS1/2 inhibitor, XAV939, and its derivatives demonstrated anti-tumor efficacy against colon [21], breast [22], and lung [23] cancers.

Given the strong implication of WNT/β-catenin signaling in the molecular pathogenesis of HCC, we rationalized that tankryase inhibitors may offer an effective means to antagonize this pathway to achieve therapeutic effects in HCC. In the current study, we investigated the anti-tumor efficacy of XAV939 and a novel nitro-substituted derivative (WXL-8), in both *in vitro* and *in vivo* models of HCC, and demonstrated that tankyrase inhibition is a feasible approach in the treatment of HCC.

## RESULTS

### TNKS1 and TNKS2 mRNA and protein levels in human HCC tumors

**Figure 1:**
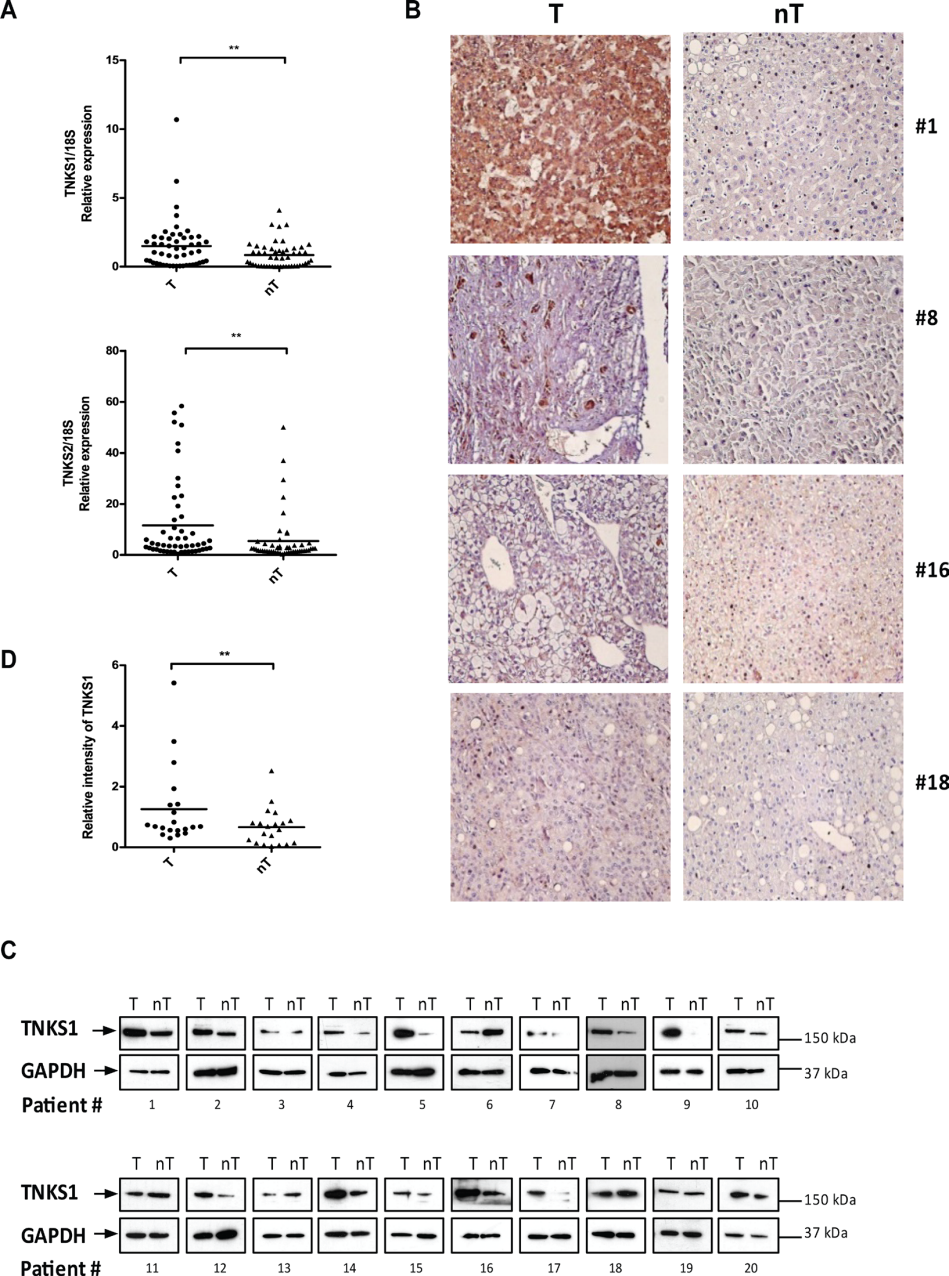
TNKS1 and TNKS2 mRNA and protein expression in HCC patient tissues **A.** Transcript levels of TNKS1 and TNKS2 is higher in tumor (T) compared to matched adjacent non-tumor (nT) tissues from 29 HCC patients, as measured by semi-quantitative real-time PCR. The amount of TNKS1 transcript was normalized with 18s RNA to control. **B.** IHC evaluation of TNKS1/2 proteins in human HCC tumors (T) compared to matched adjacent non-tumor liver (nT). The magnification used was x40 for all images. **C.** Over-expression of TNKS1 protein in human HCC tumors (T) compared to matched adjacent non-tumor liver (nT) based on Western blot analysis. **D.** The signal intensities of Western blots were quantified and shown as T to nT ratios of relative TNKS1 intensity (normalized with that of GAPDH).

To determine the clinical significance of TNKS1 or TNKS2 in HCC, we first measured their mRNA expression levels in biopsies obtained from 29 HCC patients, in comparison to matched adjacent non-tumor tissues. The mRNA expression levels of both TNKS1 and TNKS2 were significantly elevated (*P* < 0.05) in HCC tumors compared to their matched non-tumor tissues (Figure 1A). This was verified at the protein level, where we observed enhanced immunohistochemical (IHC) staining for TNKS1/2 in representative pairs of HCC and matched non-tumor liver sections from four HCC patients (Figure 1B). We further detected TNKS1 and TNKS2 using Western blot in 20 pairs of HCC and matched non-tumor liver extracts, and observed elevated TNKS1 expression in 16 of 20 HCC patients (80%) (Figure 1C). TNKS2 was undetectable using Western blot. The differential expression of TNKS1 protein in HCC and adjacent non-tumor liver tissues was statistically significant (Figure 1D) (*P* < 0.05).

### Suppression of TNKS1 and TNKS2 inhibits *in vitro* proliferation of HCC cells

**Figure 2:**
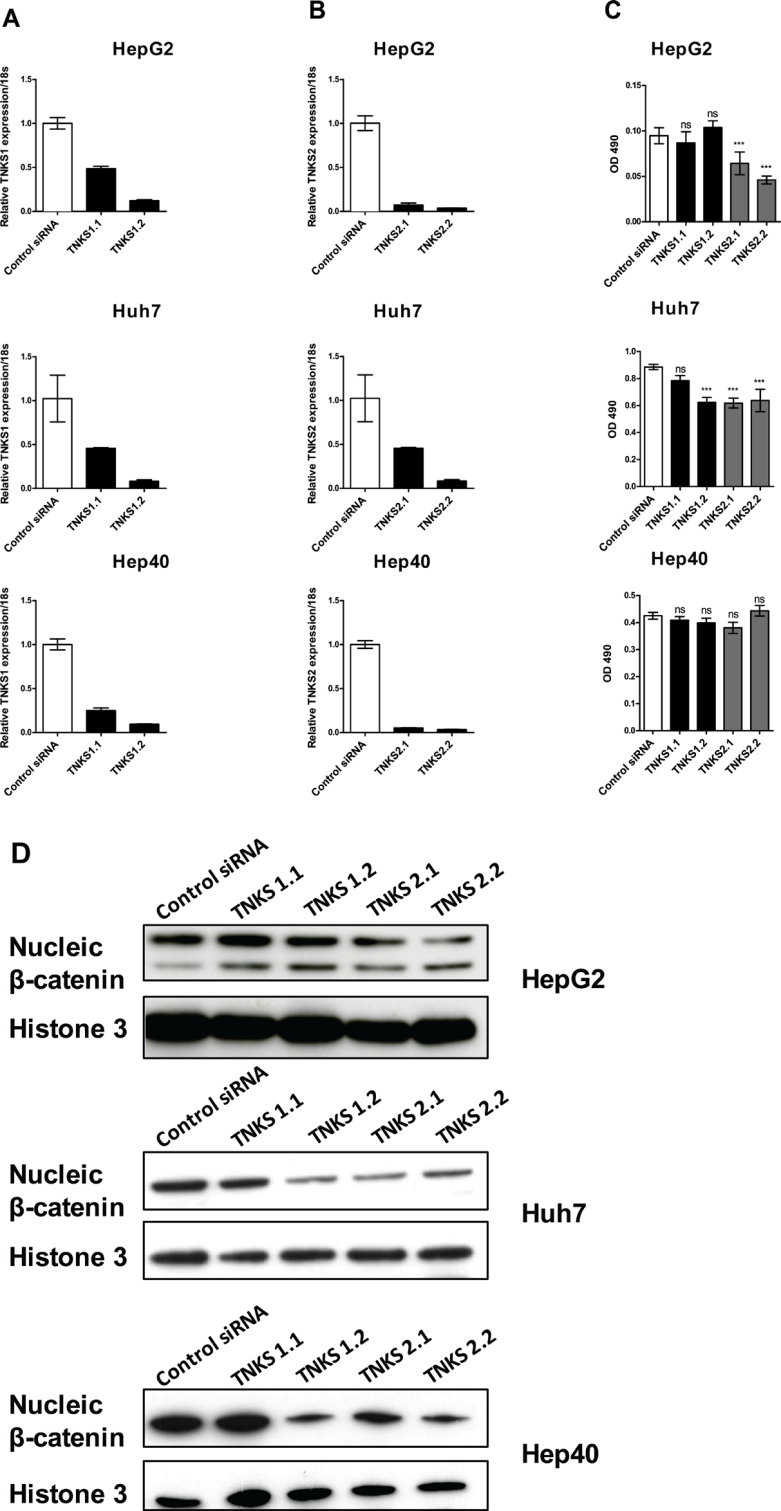
Transient knockdown of TNKS1 and TNKS2 inhibits WNT/β-catenin signaling in HCC cell lines Two independent siRNAs targeting TNKS1 (TNKS1.1 and TNKS1.2) or TNKS2 (TNKS2.1 and TNKS2.2), or a non-targeting control (Control siRNA), were transiently transfected into HepG2, Huh7, and Hep40 cells. Transcript levels of **A.** TNKS1 and **B.** TNKS2 in all three cell lines, measured by qPCR, are shown 48 hours after transient transfection with respective siRNAs. **C.** Anti-proliferative effects of TNKS1 and TNKS2 knockdown were measured by cell viability assay 3 days after transient transfection. Error bars represent standard deviations of three experiments in triplicates; *p ≤ 0.05, **p ≤ 0.01. **D.** Nuclear extracts from all three cell lines were harvested 3 days after transient transfection, and nuclei β-catenin levels were detected by Western Blotting. Histone 3 was used as nuclear protein loading control.

To validate the therapeutic potential of targeting TNKS1 and TNKS2, we first used a RNA interference approach to knockdown TNKS1 and TNKS2 in three commonly used HCC cell lines (HepG2, Hep40, and Huh7) and examined the effects on cell proliferation. Using two independent siRNAs for each target (TNKS1.1 and TNKS1.2; TNKS2.1 and TNKS2.2), we observed successful knockdown of the respective target (Figure 2A for TNKS1 and Figure 2B for TNKS2), and correspondingly significant reductions in cell proliferation in HepG2 and Huh7 cells only (Figure 2C). We observed concomitant decreases in the protein levels of nuclear β-catenin in all three cell lines after transient knockdown of either TNKS1 or TNKS2, in comparison to the control siRNA (Figure 2D). Suppression of TNKS2 (compared to TNKS1) caused greater reductions in nuclear β-catenin levels. Despite a decrease in nuclear β-catenin levels in Hep40 cells, no corresponding decrease in cell proliferation was observed.

### Tankyrase inhibitors XAV939 and WXL-8 inhibit *in vitro* proliferation of HCC cell lines

**Figure 3:**
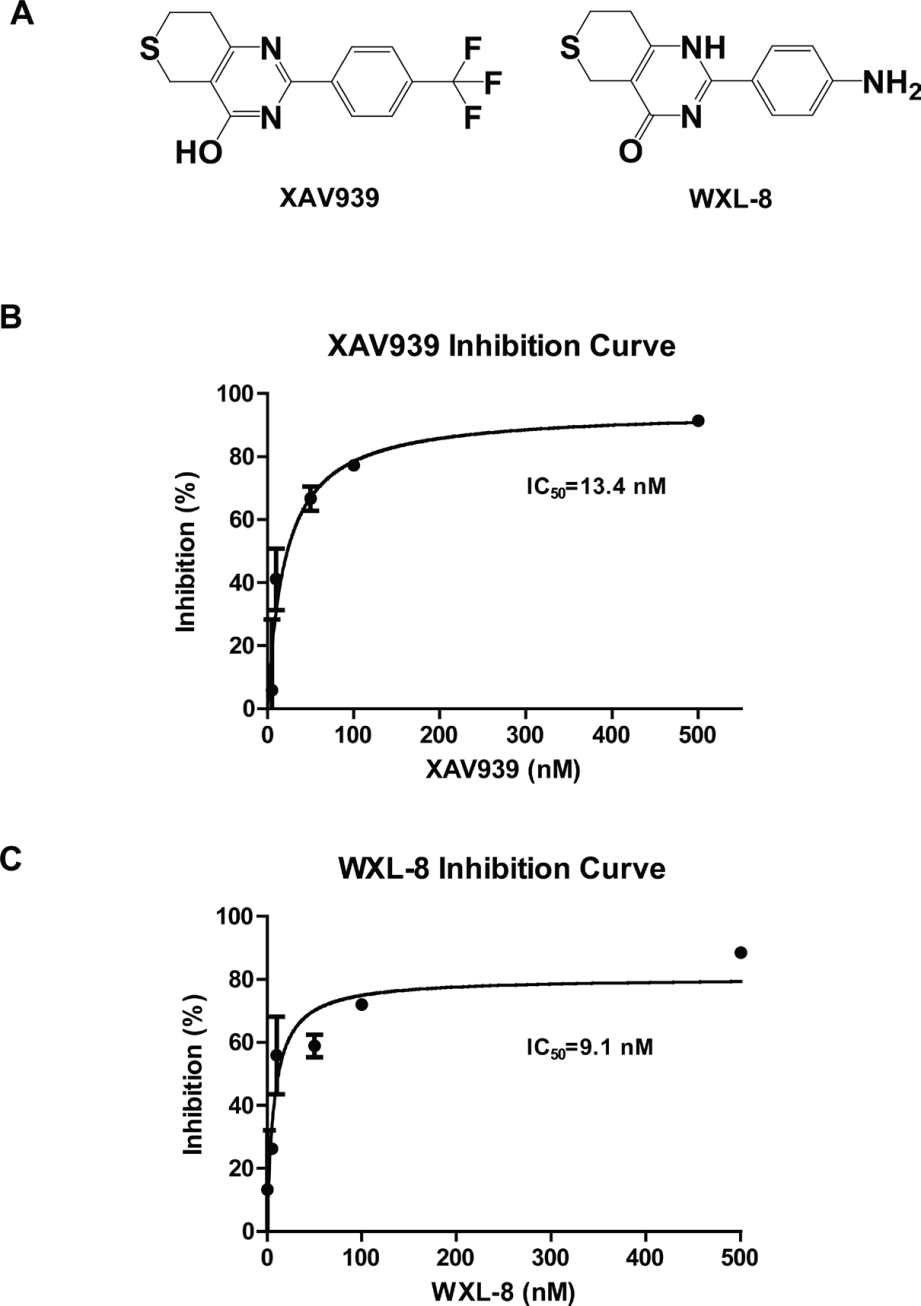
XAV939 and its derivative WXL-8 inhibit TNKS1 enzyme activity **A.** Chemical structures of XAV939 and WXL-8. Inhibitory effects of **B.** XAV939 and **C.** WXL-8 on TNKS1 enzyme activity using a colorimetric assay.

Using XAV939 as the lead compound, we synthesized a nitro-substituted derivative, named WXL-8 (Figure 3A; Supplementary Figure 1). Using the TNKS1 colorimetric enzyme activity assay, we confirmed that both compounds are effective inhibitors of TNKS1, with IC_50_s of 13.4 nM for XAV939 and 9.1 nM for WXL-8 (Figures 3B and 3C).

**Figure 4:**
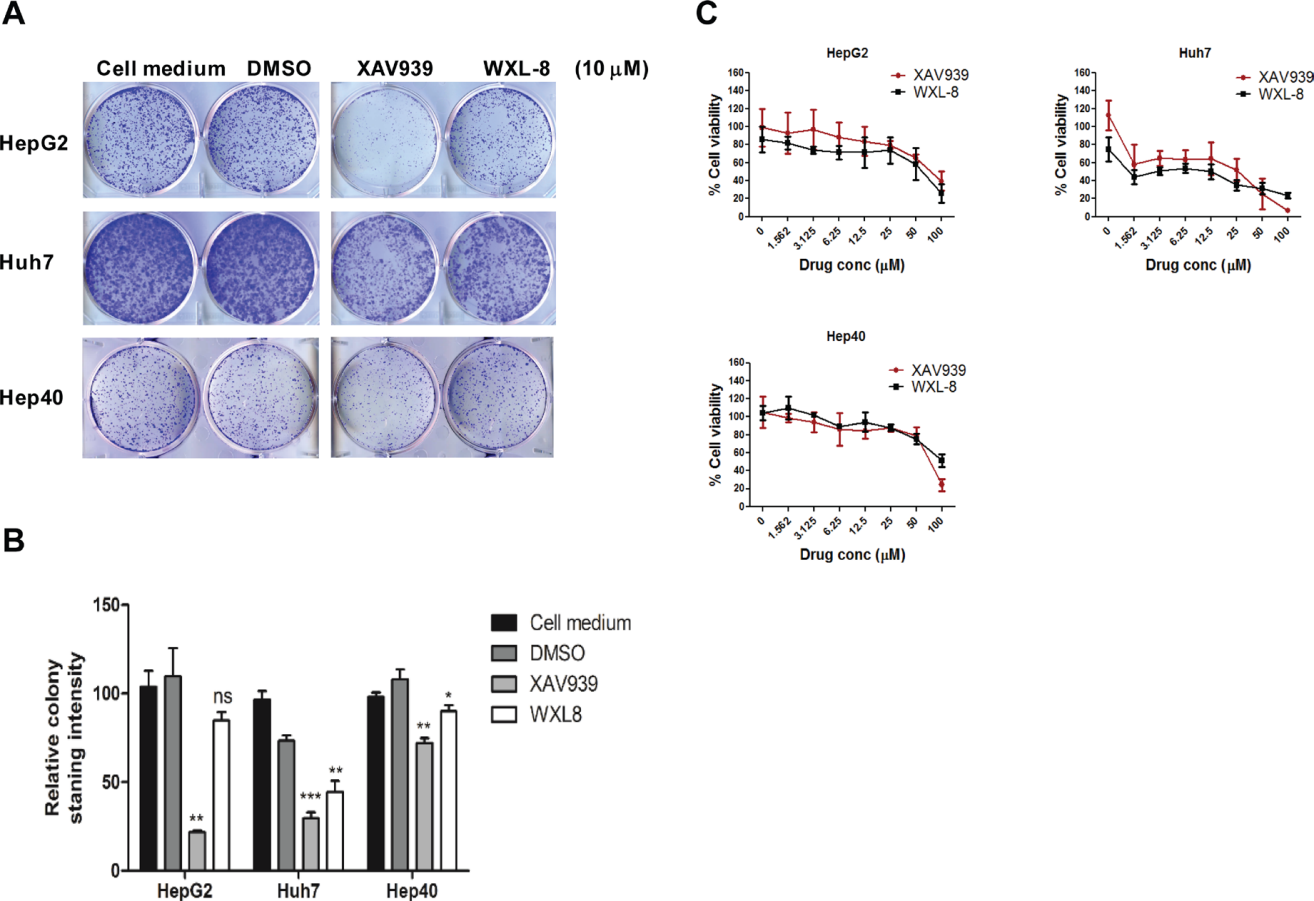
XAV939 and WXL-8 inhibit HCC cell proliferation *in vitro* **A.** Representative images from colony-forming assays of HepG2, Huh7, and Hep40 cells treated with 10 μM of XAV939 or WXL-8. **B.** Relative quantification of colony formation determined by solubilizing cell-associated dye in DMSO and measuring the OD_580nm_ of the dye–DMSO solution. Statistical comparative analyses between DMSO and drug-treated groups in three cell lines: *p < 0.01; **p < 0.001; ***p < 0.0001; n.s., not significant. **C.** Inhibition of cell viability by XAV939 and WXL-8 in HepG2, Huh7, and Hep40 cells following 7 days of drug treatment. Data are presented as mean ± SD (error bars), and expressed as % viability compared to the DMSO control group. Three independent experiments were done, each in triplicates.

We next evaluated the anti-tumor properties of XAV939 and WXL-8 using the colony formation assay in HepG2, Huh7, and Hep40 cells. Compared to cells in growth medium alone or cells treated with vehicle control (DMSO), cells treated with 10 μM of either XAV939 or WXL-8 (for 10 days) showed reductions in colony formation ability, with XAV939 having more marked effects than WXL-8 (Figures 4A and 4B). Consistent with our RNA interference data, HepG2 and Huh7 cells were also more sensitive to the growth inhibitory effects of these TNKS inhibitors.

To validate our hypothesis that tankyrase inhibition using small molecule inhibitors (XAV939 and WLX-8) is a feasible therapeutic approach for HCC, we further tested their ability to inhibit HCC cell proliferation using three HCC cell lines, which represent different molecular classes of the WNT/β-catenin pathway: HepG2 cells (which carry the truncated β-catenin mutation, partial exon 3 deletion) [24], Huh7 cells (which carry wild-type β-catenin) [9], and Hep40 cells (β-catenin status unknown, but responsive to WNT3A stimulation [data not shown]). Treatment with 2-fold dilution series of each drug, ranging from 1.56 μM to 100 μM, showed that the inhibitory effects on cell proliferation were dose-dependent in all three cell lines, with the strongest effects in Huh7 cells for both compounds (Figure 4C and Table 1). Our data suggest that tankyrase inhibition can lead to cell growth inhibition regardless of β-catenin mutation status.

**Table 1:** IC_50_ calculated for both XAV939 and WXL-8 in HCC cell lines

HCC cell line	XAV939 IC_50_ (μM)	WXL-8 IC_50_ (μM)
**HepG2**	80.71±9.33	59.79±6.99
**Huh7**	25.29±3.98	11.84±6.89
**Hep40**	52.75±4.47	87.99±8.39

### Tankyrase inhibitors XAV939 and WXL-8 stabilize AXIN1 and AXIN2 protein levels in human HCC cell lines

**Figure 5:**
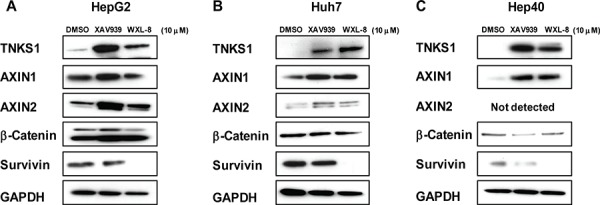
Tankyrase inhibition stabilizes AXIN levels and decreases β-catenin in human HCC cell lines **A.** HepG2, **B.** Huh7, and **C.** Hep40 cells were treated for 24 hours with 10 μM XAV939, 10 μM WXL-8, or DMSO as control, and whole cell extracts isolated for Western blotting using anti-TNKS1, anti-AXIN1, anti-AXIN2, anti-β-catenin, and anti-survivin. GAPDH was used as the loading control.

To determine whether tankyrase inhibitors interrupt the parsylation-mediated process for destabilizing AXIN protein levels, HCC cells were treated with XAV939 or WXL-8 at 10 μM, and AXIN1 and AXIN2 protein levels were detected by immunoblotting. Both AXIN1 and AXIN2 protein levels were increased following treatment with each drug, compared to cells treated with DMSO alone (Figure 5A). Expectedly, β-catenin levels were decreased in the treated cells, compared to the DMSO-treated cells for all three cell lines (Figure 5A). The protein levels of survivin, a classic downstream transcriptional target of β-catenin [25], was reduced in the XAV939-treated cells, and completely abolished in cells treated with WXL-8, indicating that both inhibitors were able to inhibit the WNT/β-catenin pathway through their effects on AXIN1, AXIN2, and β-catenin. In addition, the levels of TNKS1 were increased following these drug treatments in all three cell lines, suggesting that the tankyrase inhibitors led to stabilization of TNKS1, possibly due to the known ability of tankyrases to auto-regulate their own expression [18].

### Treatment with XAV939 and WXL-8 inhibit β-catenin/TCF4 transcriptional activity

**Figure 6:**
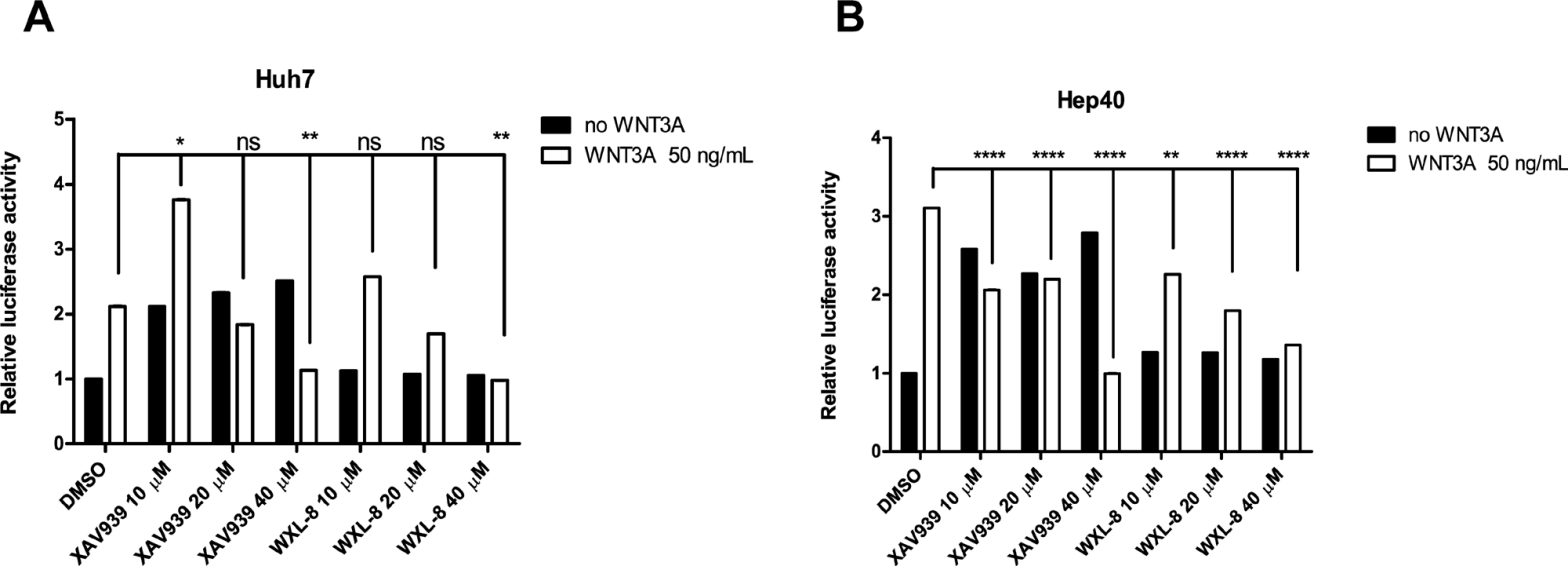
Tankyrase inhibition antagonizes canonical WNT/β-catenin signaling in human HCC cell lines XAV939 and WXL-8 attenuate TOPflash activity in **A.** Huh7 cells and **B.** Hep40 cells. TOPflash and the Renilla luciferase reporter (control) were co-transfected into respective cell lines, and incubated with XAV939 or WXL-8 at the indicated concentrations, followed by 4 hours incubation with or without rhWNT3A (50 ng/mL). The TOPflash luciferase construct activity was measured following 16 hours co-treatment with XAV939 or WXL-8. Luciferase activity was normalized to TK Renilla concentrations in each sample and compared to vehicle control. Statistical comparative analyses between DMSO and drug-treated groups in two cell lines: *p < 0.01; **p < 0.001; ****p < 0.0001; n.s., not significant.

To investigate whether tankyrase inhibitors can inhibit WNT/β-catenin signaling, we used the TOPflash reporter assay as a means to quantify perturbations in WNT/β-catenin signaling in response to treatments with XAV939 or WXL-8. Since the HepG2 cells bear a *CTNNB1* truncation mutation, making it insensitive to exogenous rhWNT3A stimulation, this cell line was not assessed. Two cell lines Huh7 (Figure 6A) and Hep40 (Figure 6B) that responded to rhWNT3A stimulation were used for this experiment. Pre-stimulation with rhWNT3A (50 ng/mL) alone increased luciferase activity in both cell lines. When the pre-stimulated cells were subsequently treated with 10, 20, or 40 μM of XAV939 or WXL-8, a dose-dependent decrease of luciferase activity was observed for both compounds (Figures 6A and 6B). These results indicate that both compounds can inhibit the β-catenin/TCF4 transcriptional activity, congruent with the decreased levels of β-catenin accumulation observed in the nucleus, which thereby decreased the formation of β-catenin/TCF complexes.

### Tankyrase inhibitors XAV939 and WXL-8 inhibit growth of subcutaneous HepG2 xenografts

**Figure 7:**
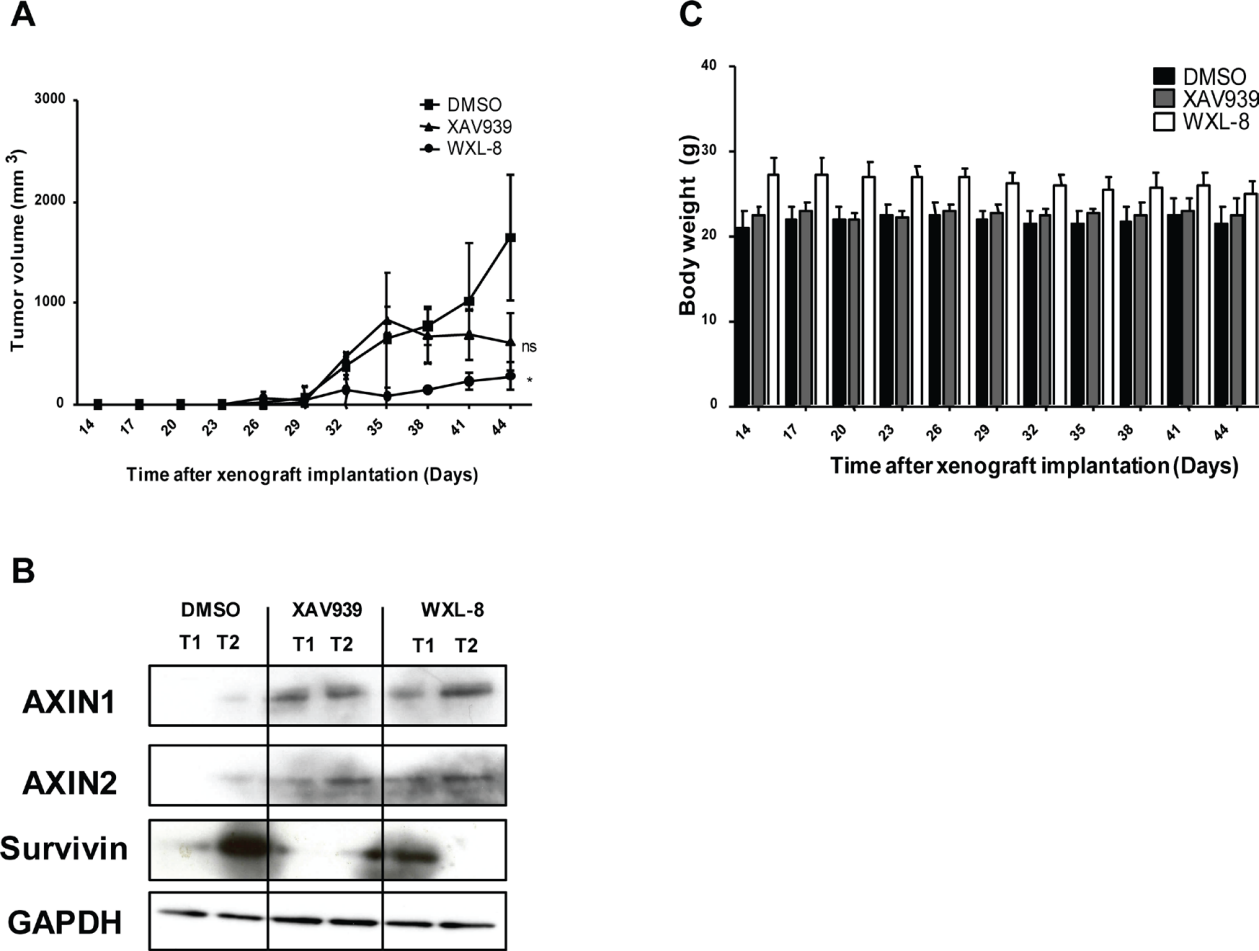
XAV939 and WXL-8 inhibits growth of a HepG2-derived liver tumor xenograft model Animals were randomized into three groups (*n* = 5 each): DMSO vehicle control group, XAV939 group, and WXL-8 group. Intratumoral injections were given every 3^rd^ day for 3 weeks. **A.** Tumor volumes were monitored during the 3-week treatment period. **B.** XAV939 and WXL-8 enhanced AXIN protein levels in HepG2 xenograft tissues. Representative Western Blot images of AXIN1 and AXIN2 protein levels in two random tumors (T1 and T2) after 3 weeks of treatment with each compound and DMSO vehicle control. Survivin protein levels were reduced. GAPDH was used as a loading control. **C.** Total body weight of tumor-bearing mice over the 3-week treatment period.

The hydrophobic nature of both XAV939 and WXL-8 precluded intravenous or intraperitoneal administration. When HepG2 xenografts have grown to approximately equal luminescence signals, mice were randomized into three groups (*n* = 5 each) for intra-tumor injection with 20 mg/kg of XAV939, WXL-8, or vehicle control, every three days for three weeks. Treatment with either XAV939 or WXL-8 caused reductions in tumor volume compared to vehicle control group, although significance was only observed for WXL-8 (Figure 7A). WXL-8 exerted greater anti-tumor activity than XAV939, which is consistent with *in vitro* data that this compound had greater anti-proliferative effect (Table 1), and also caused greater reduction in β-catenin levels (Figure 5). Also consistent with *in vitro* observations, we detected enhanced levels of AXIN1 and AXIN2 proteins, and reduced levels of survivin, in xenograft tissues treated with XAV939 or WXL-8, compared to untreated xenografts (Figure 7B). Monitoring of animal weight over the treatment period revealed no significant changes in body weight (Figure 7C), implying that XAV939 and WXL-8 did not exert major systemic toxicity when local tumor injection is applied.

## DISCUSSION

In HCC, the frequently hyperactivated WNT/β-catenin signaling pathway represents a promising target for molecular therapies. Currently, only a few of chemicals that have been developed to target this pathway have entered into pre-clinical trials, and none of these chemicals have advanced to the late clinical trial stage [26]. Multiple key factors along the WNT/β-catenin signaling cascade represent potential therapeutic targets [27]; here we provide initial evidence to support that the TNKS enzymes are feasible targets with clinical relevance in HCC.

In this study, we confirmed the predominant over-expression of TNKS1 mRNA and protein levels in human HCC tumors compared to adjacent non-tumor liver tissues, whereas TNKS2 is elevated only at mRNA level. Knockdown of TNKS1 and TNKS2 in HCC cell lines by RNA interference resulted in inhibition of cell proliferation and reduction in nuclear β-catenin protein levels, validating the anti-tumor potential of inhibiting these enzymes in the WNT/β-catenin pathway. Using XAV939 as the prototype small molecule tankyrase inhibitor, we demonstrated that pharmacological inhibition of TNKS1 achieved similar effects as TNKS1 knockdown in HCC cell lines. Mechanistically, decreased cell proliferation caused by XAV939 was associated with increased AXIN1 and AXIN2 protein levels, decreased β-catenin protein levels, and decreased β-catenin/TCF4 transcriptional activity. A nitro-substituted derivative of XAV939, WXL-8, exerted similar anti-tumor effects in HCC cell lines.

The cell lines used in this study represent different molecular subclasses of HCC, such as those with mutant or wild-type *CTNNB1*. We show that regardless of the mutation status of *CTNNB1*, AXIN stabilization *via* inhibition of tankyrases can still effectively inactivate WNT/β-catenin signaling, suggesting that AXIN function may represent a key regulatory node in the WNT/β-catenin signaling cascade. Importantly, we observed that the XAV939 derivative, WXL-8, demonstrated superior anti-proliferative activity *in vitro* (compared to the parent compound), and was able to completely abolish the nuclear accumulation of β-catenin. *In vivo*, WXL-8 was also more effective than XAV939 at inhibiting the growth of subcutaneous HepG2 xenografts, although neither compound caused any significant changes in the body weight of tumor-bearing mice. Thus, we have provided the first proof-of-concept for the therapeutic use of tankyrase inhibitors in HCC.

While tankyrase inhibition appears to be a viable approach in cancer therapeutics, several groups have reported challenges with the use of XAV939 in their preclinical tumor models. For example, Bao *et al* reported that XAV939 was effective against breast cancer cell lines only under low serum conditions [22] suggesting that its anti-tumor effect may be overridden by other growth factors and signaling pathways. This may be the case for Hep40 cells, which was least sensitive to both TNKS1/2 mRNA suppression and pharmacological inhibition, compared to HepG2 and Huh7 cells. Thus, combination therapy including tankyrase inhibitors and other conventional or molecularly targeted therapy may be required to achieve clinically desirable outcome. In our study, we were able to observe anti-tumor effects under optimal serum conditions; however, *in vitro* anti-proliferative effects were only modest, also suggesting that combination therapy or a more potent tankyrase inhibitor may be necessary.

During the course of our manuscript preparation, Schultz *et al* reported the identification of a highly potent, selective, and orally active tankyrase inhibitor (NVP-TNKS656) [28]. This compound had a significantly improved IC_50_ (to nm level) and achieved good plasma and tumor exposure levels in mice, with a decrease in Axin2 mRNA levels as early as 4 hours post-treatment. However, no study has yet shown its *in vivo* anti-tumor efficacy in any tumor type. Thus, it would be of interest to evaluate the toxicity and anti-tumor efficacy of this compound (either singly or in combination with other standard of care agents such as sorafenib) in mouse models of HCC, including patient-derived xenografts.

Taken together, we demonstrate that TNKS1 and TNKS2 are clinically relevant and druggable targets for a subgroup of HCC patients. Pharmacological inhibition of these enzymes may achieve desirable anti-tumor effects *via* AXIN stabilization with subsequent effective attenuation of downstream β-catenin signaling, and offer a new approach for the treatment of multiple cancers that over express TNKS1 or TNKS2.

## MATERIALS AND METHODS

### Chemicals

XAV939 was purchased from Sigma Aldrich (St. Louis, MO). WXL-8 was synthesized according to the synthetic scheme shown in Supplementary Figure 1. The ^1^H and ^13^C NMR spectra were obtained using the Varian 100 MHz or 400 MHz magnetic resonance spectrometer respectively, and are shown in Supplementary Figures 2 and 3. High resolution mass spectrometric (MS) analyses of the compounds were performed at the Mass Spectrometry Facility at Stanford University (Palo Alto, CA), and are shown in Supplementary Figure 4. XAV939 and WXL-8 were dissolved in dimethyl sulfoxide (DMSO) at 10 mM as stock solutions and stored at −20°C for subsequent use in cell-based experiments.

### Cell lines

The human HCC cell lines HepG2 and Hep40 were obtained from ATCC (Manassas, VA), and the Huh7 cell line was a gift from Dr. Mark Kay (Stanford University, CA). The cell lines were maintained at 37°C in a humidified atmosphere (5% CO_2_) in the following media types, supplemented with 10% fetal bovine serum, 100 μg/mL penicillin and 100 μg/mL streptomycin: Eagle's Minimum Essential Media (for HepG2), Dulbecco's Modified Eagle's Medium (DMEM; for Huh7 and Hep40). All media and supplements were obtained from Invitrogen (Carlsbad, CA).

### RNA isolation and semi-quantitative real-time PCR

Paired HCC and adjacent non-tumor liver tissues were obtained from 29 HCC patients who underwent liver resection at Stanford Hospital, Stanford University. This study was carried out with pre-approval from the Stanford University Institutional Review Board, and informed consent for study participation was obtained from all patients prior to liver resection. Total RNA was extracted from liver tissues and cultured cells by using the RNeasy Mini Kit (Qiagen, Valencia, CA), and 2 μg were reverse transcribed by using the High-Capacity cDNA Reverse Transcription Kit (Invitrogen, Carlsbad, CA). Real-time PCR analysis was then carried out using the *Taq*Man assays for TNKS1 (Hs00186671_m1), TNKS2 (Hs00228829_m1), and 18S (Hs99999901_s1, used as the endogenous control) (Applied Biosystems Inc., Foster City, CA) in a 7500 Fast Real-Time PCR System (Applied Biosystems Inc., Foster City, CA). The relative mRNA levels were calculated by the comparative threshold cycle method. All samples were run in triplicates for each experiment.

### Protein extraction and immunoblotting

Total protein from patients, animal xenograft tissues and HCC cell lines were extracted with the T-PER Tissue Protein Extraction Reagent supplemented with protease inhibitor (all from Thermo Fisher Scientific Inc., Rockford, IL). After centrifugation at 13,000 rpm for 15 minutes at 4°C, supernatants were collected and total protein concentration measured by BCA Protein Assay Kit (Pierce, Rockford, IL). Nuclear protein was extracted using the NE-PER Extraction Kit (Pierce, Rockford, IL) following the manufacturer's protocol. The total protein lysates (10 μg for HCC cells and 20 μg for patient samples) or nuclear extracts (5 μg) were mixed with loading buffer, resolved on sodium dodecyl sulfate-polyacrylamide gels, and electrotransferred onto nitrocellulose membranes. The membrane was blocked for 1 hour at room temperature in 5% milk. Membranes were incubated with desired primary antibodies (listed below) overnight at 4°C, after which the appropriate horseradish peroxidase-conjugated secondary antibodies (listed below) were added and allowed to incubate for 2 hours at room temperature. The immunoreactive complexes were detected by using the Super-Signal West Pico Chemiluminescent and or West Femto Maximum Sensitivity substrate (Thermo Fisher Scientific Inc., Rockford, IL), according to the manufacturer's protocols. The primary antibodies used were: 1:250 TNKS 1/2 ( H-350, Santa Cruz Biotechnology, Santa Cruz, CA), 1:500 anti-TNKS 1 (AF7116; R&D Systems, Minneapolis, MN), 1:1000 anti-AXIN1 and 1:1000 anti-AXIN2 (C95H11 and D48G4, respectively; Cell Signaling Technology Inc., Danvers, MA), 1:1000 anti-β-catenin (sc-7963; Santa Cruz Biotechnology, Santa Cruz, CA), and 1:1000 anti-GAPDH as loading control (Santa Cruz Biotechnology, Santa Cruz, CA); 1:1000 anti-histone 3 as the nuclear protein loading control (ab21054; Abcam, Cambridge, MA). The secondary antibodies used were: 1:2500 goat anti-rabbit and 1:2500 goat anti-mouse (sc-2006 and sc-2005 respectively; Santa Cruz Biotechnology, Santa Cruz, CA), and 1:2500 anti-sheep (HAF016; R&D Systems, Minneapolis, MN). Protein band intensities were quantified using ImageJ software and normalized to the internal loading control GAPDH.

### Immunohistochemistry of HCC patient samples

HCC and adjacent non-tumor samples were embedded in paraffin and sectioned at 5 μm thickness for immunohistochemical staining. Slides were pretreated with citrate buffer (10 mM sodium citrate, pH 6.0) for heat-induced epitope retrieval. Endogenous peroxidase activity was blocked with 3% H_2_O_2_. Non-specific protein binding was blocked with 10% goat serum for 1 hour. Primary TNKS 1/2 antibodies at 1:50 dilution were added to the sections and incubated at 4°C overnight. Secondary antibodies were added to the sections and incubated at room temperature for 1 hour. Sections were developed using Dako EnVision system and were then counterstained with hematoxylin (Dako, Glostrup, Denmark). Images were viewed with a fluorescence microscope (Nikon Eclipse 80i, Nikon Corporation, Tokyo, Japan).

### Transient siRNA transfection

Knockdown experiments using siRNA were done using two independent pre-validated siRNAs that targeted human TNKS1 or TNKS2, and a Silencer Negative Control No. 1 siRNA (used as control) (Invitrogen, Carlsbad, CA). HepG2, Huh7, and Hep40 cells were seeded in 96-well plates, with 4000 cells in 200 μL of media per well. Target-specific and control siRNAs were mixed separately with Lipofectamine RNAiMAX Transfection Reagent (Invitrogen, Carlsbad, CA) to obtain a final concentration of 25 nM and incubated at room temperature for 15 minutes before adding to the cells. At 72 hours post-transfection, cell viability was assayed as described below after removal of cell media.

### Colony formation assay

Each of the HCC cell lines was seeded in 6-well plates, with 5000 cells in 2 mL of media per well. The cells were then treated with either 0.1% DMSO alone (used as vehicle control) or with 10 μM of XAV939 or WXL-8 (dissolved in DMSO) and incubated for 10 days, until colonies became sufficiently large to quantify. The media and compounds were replaced on days 3, 6 and 9. On day 10, the cells were washed once with 1x phosphate buffered saline (PBS), fixed in ice-cold methanol for 10 minutes, and stained with 0.5% crystal violet (in 25% methanol) for 10 minutes at room temperature. After rinsing with double-distilled water and drying at room temperature, images of the colonies were obtained using an Epson scanner. Each treatment was evaluated in triplicates, and representative images are shown. For relative quantification of colony formation, we determined the colony staining intensity by solubilizing the cell-associated dye in DMSO and measuring absorbance (OD_580_) of the dye–DMSO solution in a Powerwave XS microplate spectrophotometer (BioTek, Winooski, VT) [29].

### Cell proliferation assay

Each of the HCC cell lines was seeded in 96-well, clear bottom plates (BD Biosciences, Franklin Lakes, NJ), with 500 cells in 200 μL of growth media per well. The cells were then treated with either 0.5% DMSO alone (used as vehicle control) or with XAV939 or WXL-8 in a 2-fold dilution range (from 100 μM to 1.562 μM). After 7 days of incubation, the media and compounds were removed and replaced with 100 μL of fresh growth media and 20 μL of CellTiter-96 AQueous One Solution Reagent (Promega, Madison, WI). After incubation for 2 to 4 hours, absorbance was measured at 490 nm using the Powerwave XS microplate spectrophotometer (BioTek, Winooski, VT).

### TOPflash luciferase reporter assay

The Huh7 and Hep40 cells were plated in 24-well plates, at a density of 2 × 10^5^ cells in 500 μL media per well. The co-transfection solution (100 μL per well) was made by mixing 750 ng of TOPflash reporter plasmid or 750 ng of FOPflash reporter plasmid with 50 ng TK-Renilla plasmid (for a total of 800 ng) and 2 μL of Lipofectamine 2000 Transfection Reagent (Invitrogen, Carlsbad, CA). The transfection mixture was incubated at room temperature for 15 minutes. Meanwhile, the growth media were removed from each well and 400 μL of serum-free DMEM was added before the 100 μL transfection mixture was added. After 6 hours of incubation, media plus transfection mixture were removed and replaced with fresh serum-free media containing XAV939 or WXL-8 at a final concentration of 10, 20, or 40 μM supplemented with 50 ng/mL of rhWNT3a ligand (R&D Systems, Minneapolis, MN). DMSO was used as the vehicle control. At 24 hours post-drug treatment, the cells were lysed in Passive Lysis Buffer (Promega, Madison, WI) and luciferase activities were measured using the Dual-Luciferase Assay System (Promega, Madison, WI) and a Veritas Microplate Luminometer (Turner Biosystems, Promega, Madison, WI). Firefly luciferase activity was normalized to activity of Renilla Luciferase. Control conditions were set to one, and fold activities are shown relative to this. All experimental conditions were assessed in triplicates, and the experiments were repeated at least three times.

### Xenograft mouse model and drug treatment

Animal studies were carried out in compliance with all federal and local institutional rules for the conduct of animal experiments. To generate subcutaneous xenografts, 1 × 10^6^ HepG2 cells were suspended in 100 μL of Dulbecco's PBS (Invitrogen, Carlsbad, CA) and injected subcutaneously near the right forelimb of adult (4–6 weeks old) male NOD-scid-gamma (NSG) mice (Charles River Laboratories Inc., Cambridge, MA). The HepG2 cells were transduced with self-inactivating lentivirus that carried a ubiquitin promoter driving a trifusion reporter gene, which harbored reporter genes for detection by bioluminescence (firefly luciferase [*Fluc*]), fluorescence (*mrfp* or *egfp*), and positron emission tomography (*ttk*), at a multiplicity of infection of 5, as reported previously [30]. Optical imaging was done at day 7 (Supplementary Figure 5) and day 14 (Supplementary Figure 6) to ensure equal tumor-associated luciferase activity within each mouse. For imaging, mice were administered firefly luciferin (150 mg/kg; Xenogen Corp., Alameda, CA) by intraperitoneal injection. Mice were then randomized into three groups (*n* = 5 each), and given intra-tumor injections of DMSO (control), 20 mg/kg of XAV939, or 20 mg/kg of WXL-8 every 3 days for 3 weeks. Tumor growth was monitored weekly by bioluminescence imaging after intraperitoneal injection of D-luciferin (Xenogen IVIS system; Caliper Life Sciences, Hopkinton, CA). Additionally, tumor size was measured with a caliper before and after treatments, and the tumor volumes were calculated using the formula: [W(2) × L]/2. The whole body weight of each animal was also monitored during the course of treatment.

### Statistical analyses

All cell-based experiments were independently repeated at least three times, and representative experiments are shown. All quantitative data are reported as means ± SD. Paired *t*-test was used to calculate statistical differences between paired groups. Differences between two or more experimental groups were analyzed by one-way ANOVA with Tukey's post-hoc test. *P* values of < 0.05 were considered significant. All statistical analysis was carried out using GraphPad Prism (GraphPad Software, San Diego, CA).

## SUPPLEMENTARY FIGURES AND LEGENDS


