# Supplementary material for: Tankyrase inhibitors attenuate WNT/β-catenin signaling and inhibit growth of hepatocellular carcinoma cells
Source: Oncotarget. 2015 Jun 27;6(28):25390–401. doi: 10.18632/oncotarget.4455 (PMC4694839; doi:10.18632/oncotarget.4455)
Supplement: Supplementary file 1 [file oncotarget-06-25390-s001.pdf]

SUPPLEMENTARY FIGURES AND LEGENDS

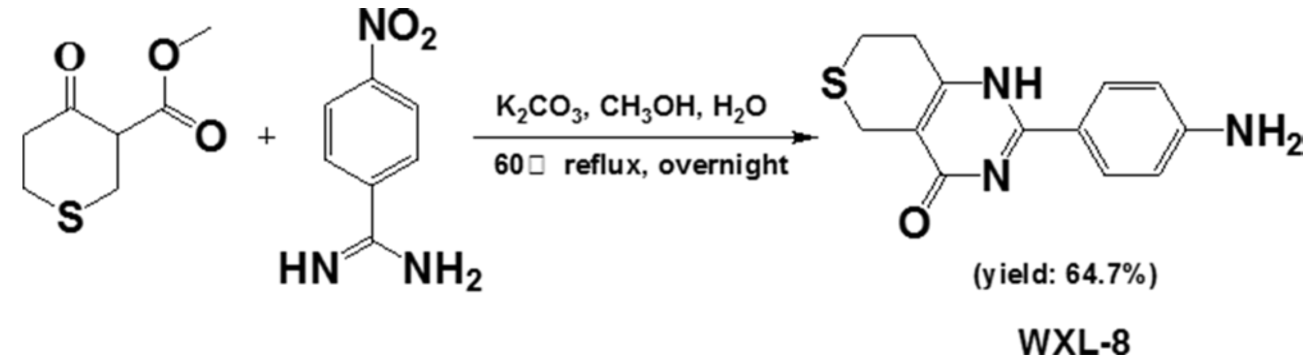

Supplementary Figure S1: Synthetic scheme of WXL-8.

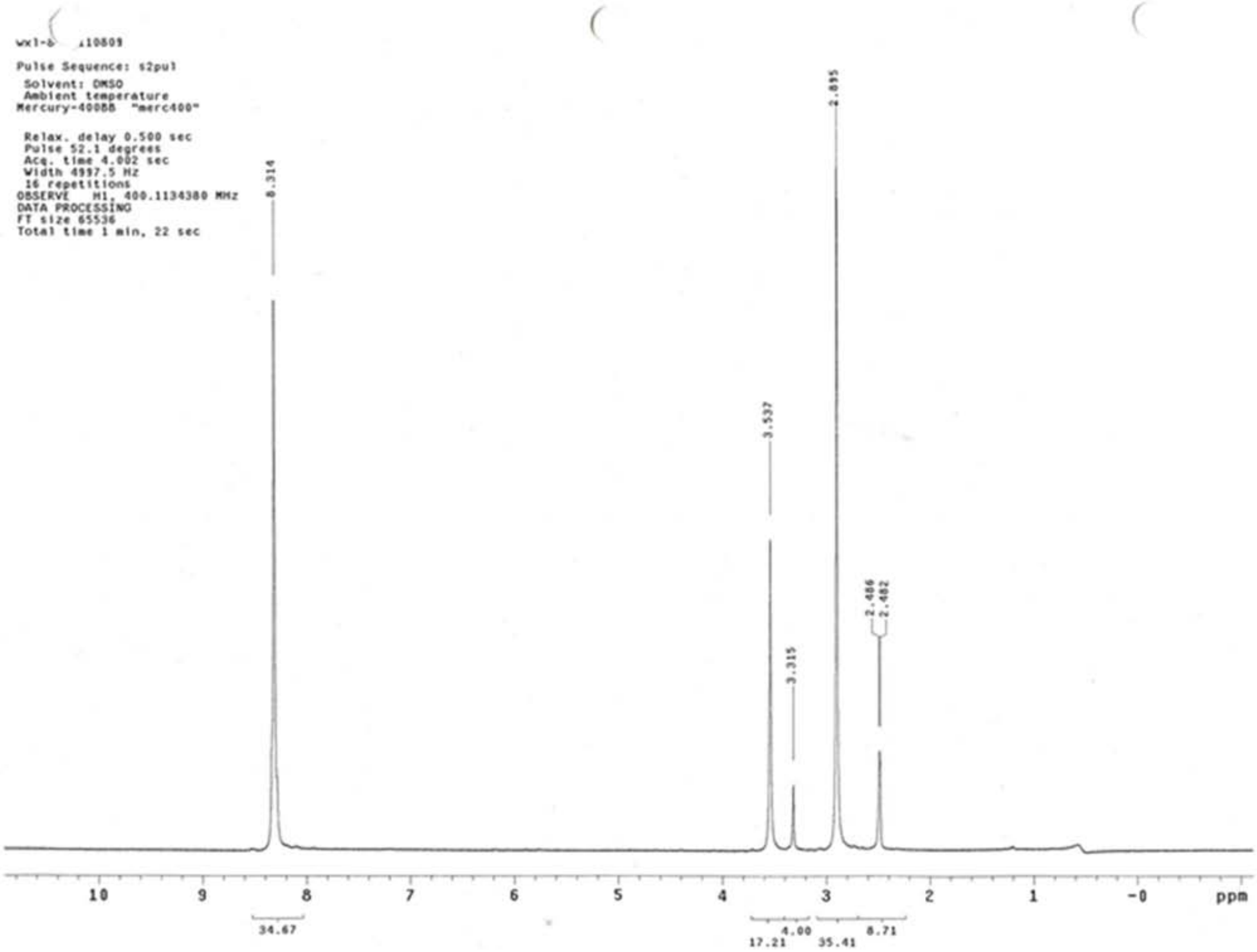

Supplementary Figure S2: ^{1}H NMR data for WXL-8.

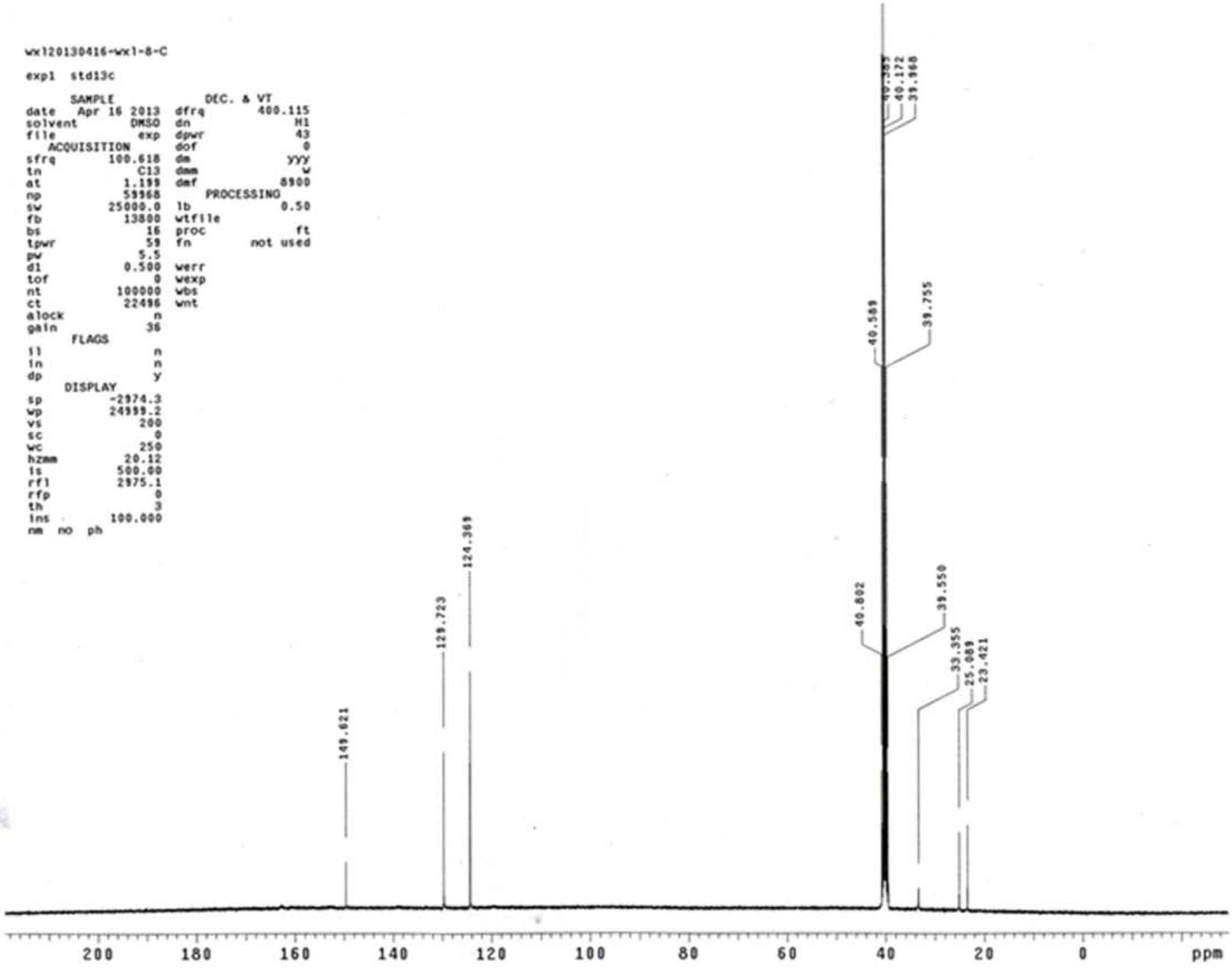

Supplementary Figure S3: ^{13}C NMR data for WXL-8.

Mass Spectrum SmartFormula Report

Analysis Info

Analysis Name	D:\Data\HRAM\130415\data\WangX_41769_WXL8_02_94_01_6859.d	Acquisition Date	4/15/2013 2:17:29 PM
Method	loopinj_pos_50_1200.m	Operator	BDAL@DE
Sample Name	WangX_41769_WXL8_02	Instrument / Ser#	micrOTOF-Q II 10292
Comment			

Acquisition Parameter

Source Type	ESI	Ion Polarity	Positive	Set Nebulizer	2.5 Bar
Focus	Not active	Set Capillary	4500 V	Set Dry Heater	250 °C
Scan Begin	50 m/z	Set End Plate Offset	-500 V	Set Dry Gas	8.0 l/min
Scan End	1200 m/z	Set Collision Cell RF	100.0 Vpp	Set Divert Valve	Source

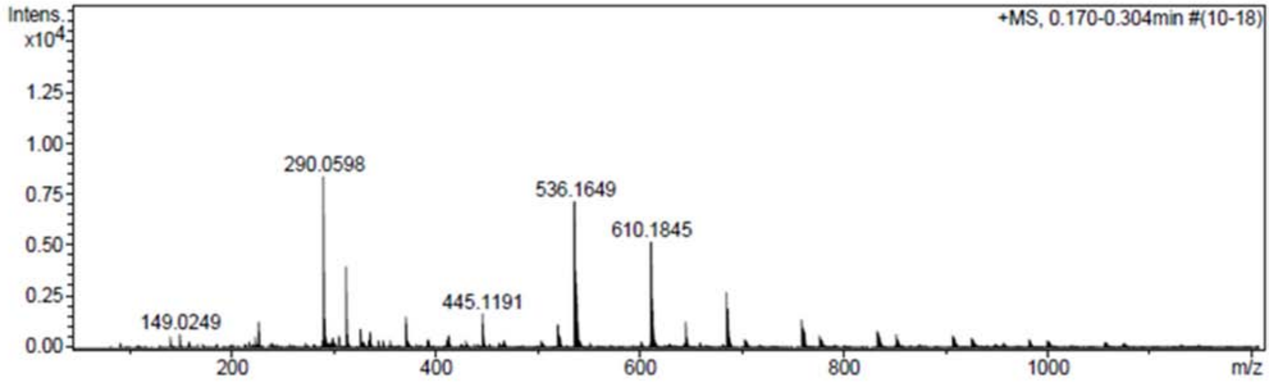

Meas. m/z	#	Formula	m/z	mSigma	err [ppm]	Score	err [mDa]	rdb	e⁻ Conf	N-Rule
290.0598	1	C 13 H 12 N 3 O 3 S	290.0594	6.1	-1.6	100.00	-0.5	9.5	even	ok
	2	C 21 H 8 N O	290.0600	42.8	0.7	48.64	0.2	18.5	even	ok
	3	C 8 H 9 N 7 Na O 4	290.0608	45.4	3.4	30.55	1.0	7.5	even	ok
	4	C 6 H 4 N 13 O 2	290.0605	46.5	2.4	34.41	0.7	11.5	even	ok
	5	C 7 H 13 N 3 Na O 8	290.0595	54.8	-1.2	31.62	-0.4	2.5	even	ok
	6	C 5 H 8 N 9 O 6	290.0592	55.5	-2.2	27.03	-0.6	6.5	even	ok

Supplementary Figure S4: High resolution mass spectrometry data for WXL-8.

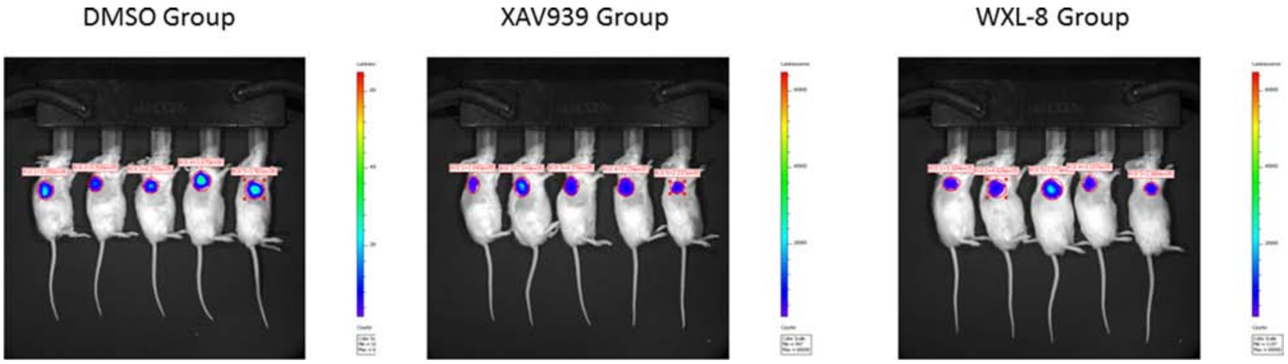

Supplementary Figure S5: Luciferase imaging of mice bearing HepG2 xenografts stably expressing a tri-fusion reporter gene, on day 7 post-inoculation.

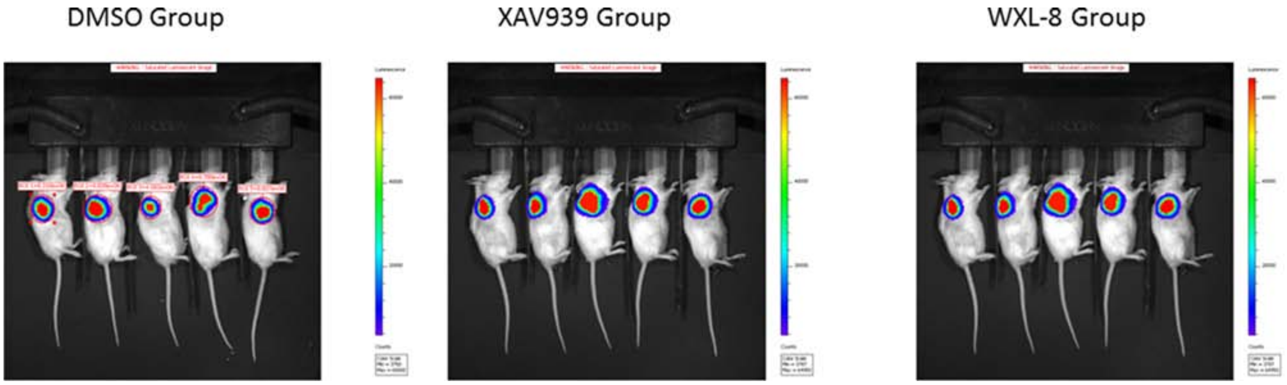

Supplementary Figure S6: Luciferase imaging of mice bearing HepG2 xenografts stably expressing a tri-fusion reporter gene, on day 14 post-inoculation.
